# Anti- Schistosomular Activity of Human Monocytes/Macrophages in Response to Interleukin-3 and Granulocyte-Macrophage Colonystimulating Factor Stimulation

**DOI:** 10.1155/S0962935194000451

**Published:** 1994

**Authors:** D. Gold, L. Nissimov, J. Lengy, Y. Keisari

**Affiliations:** Department of Human Microbiology Sackler Faculty of Medicine Tel Aviv University Tel Aviv 69978 Israel

## Abstract

Human monocytes, co-incubated for 7 days in culture with GM-CSF or IL-3 but not with IFN-γ, exerted a variable schistosotnulicidal effect on *Schistosoma mansoni* parasites when grown in 96-well round-bottomed plates but not in flat-bottomed plates. Addition of LPS or IFN-γ or both, for the last 48 h did not enhance the cidal effect. Addition of LPS but not IFN-γ to the pre-incubated cells with GM-CSF or IL-3 markedly stimulated TNF-α production by the cells but not their cidal activity. The variable cidal effects obtained with the monocytes/macrophages from different donors suggest that these effects may be genetically predetermined and are possibly linked to blood group markers or to MHC class I or II antigens.


Mediators of Inflammation 3, 329–333 (1994)

### Anti-schistosomular activity of human monocytes/macrophages in response to interleukin-3 and granulocyte–macrophage colony-stimulating factor stimulation

D. Gold,[CA] L. Nissimov, J. Lengy, and Y. Keisari

Department of Human Microbiology, Sackler Faculty of Medicine, Tel Aviv University, Tel Aviv 69978, Israel

[CA] Corresponding Author

**HUMAN monocytes, co-incubated for 7 days in culture with GM–CSF or IL-3 but not with IFN-γ, exerted a variable schistosomulicidal effect on *Schistosoma mansoni* parasites when grown in 96-well round-bottomed plates but not in flat-bottomed plates. Addition of LPS or IFN-γ or both, for the last 48 h did not enhance the cidal effect. Addition of LPS but not IFN-γ to the pre-incubated cells with GM–CSF or IL-3 markedly stimulated TNF-α production by the cells but not their cidal activity. The variable cidal effects obtained with the monocytes/macrophages from different donors suggest that these effects may be genetically predetermined and are possibly linked to blood group markers or to MHC class I or II antigens.**

**Key words**: GM–CSF, IFN-γ, IL-3, LPS, Monocyte/macrophage antischistosomular cytotoxicity, TNF-α

### Introduction

Macrophages from schistosome-infected mice have been shown to constitute efficient cytotoxic agents against freshly prepared schistosomula of *Schistosoma mansoni*.[1,2] However, since the human immune response to this parasite may not necessarily be identical to that of mice,[3] several investigations have studied the cytotoxic potential of human monocytes/macrophages in systems devoid of, or replete with, stimulants or specific antibodies.[4–7] James *et al.*[8] assessed the enhanced cytotoxic effect of human monocytes/macrophages following their stimulation with human recombinant CSF-1, human recombinant IFN-γ or a combination of both. The present work likewise deals with a similar interaction (albeit with different stimulators) between normal human monocytes or monocyte-derived macrophages and *S. mansoni* schistosomula, this following stimulation of the cells by known monocyte modulators—namely, granulocyte–macrophage colony-stimulating factor (GM–CSF), interleukin 3 (IL-3), or human recombinant interferon-γ (IFN-γ) and/or subsequently added bacterial lipopolysaccharide (LPS) or IFN-γ.

### Materials and Methods

*Reagents*: Human recombinant GM–CSF and IL-3 (courtesy of Behringwerke, Marburg, Germany) were used at concentrations of 1 000 U/ml each, after first establishing that this was the optimal concentration for stimulation of the cells. Human recombinant IFN-γ (courtesy of Drs M. Rubinstein and D. Novik, Weizmann Institute of Science, Rehovot, Israel) was added to the cell-containing wells either for the whole duration of the experiment or for the last 48 h only (at 1 000 U/ml), or as a combination of both. LPS (Sigma, St. Louis, MO, USA) was added to the cell cultures, for the last 48 h, at 10 μg/ml. $N^G$-monomethyl arginine ($N^G$MMA), a competitive inhibitor of arginine metabolism, was added to the appropriate wells at 0.1 mM for the last 48 h of incubation with the cells, concurrently with the schistosomula.

*Cells*: Mononuclear cells were obtained from normal human blood donors after Ficoll-Hypaque separation of the PBS-suspended buffy coats. For later introduction into wells of flat-bottomed plates, the cells were kept overnight in RPMI 1640 + 10% NBS at 4°C, following which the platelet-rich supernatant was decanted and the sediment suspended in RPMI 1640 + 2% FBS. Viability of the cells was assessed by trypan blue exclusion, then their concentration was adjusted to $3 \times 10^7$/ml and 0.1 ml aliquots of the cell suspensions were introduced into wells of flat-bottom microtitre plates. After 1 h incubation at 37°C in air containing 7.5% $CO_2$, the wells were gently rinsed three times with warm (37°C) Earle's BSS, then overlayed with RPMI 1640 medium containing 5% FBS to which 300 μg/ml glutamine, 20 mM HEPES and antibiotics (200 U/ml penicillin and 200 μg/ml streptomycin) were added. With a plating efficiency of about 15%, this resulted in approximately $4.5 \times 10^5$ adherent cells/well. In initial experiments, some wells were checked for monocyte content by esterase activity, and more than 90% of them proved

© 1994 Rapid Communications of Oxford Ltd to be esterase positive.

For introduction into round-bottomed wells of microplates, monocytes were first freed of other cells by inducing them to adhere to the bottoms of plastic tissue culture flasks and washing off the non-adherent cells; to this end, $5 \times 10^6$/ml of the cell suspensions were incubated for 1 h at 37°C in RPMI 1640 + 2% FBS, following which the adherent cells were washed three times with warm Earle's BSS, thereby discarding practically all non-adherent cells. The adherent cells were then incubated overnight at 37°C and 7.5% $CO_2$, in RPMI 1640 + 10% FBS. This procedure, modified from Treves *et al.*,[9] as well as repeated rinsing with cold Earle's BSS, caused detachment of most of these cells. Detached cells were collected, washed twice, counted and checked for viability, and adjusted to $3 \times 10^6$/ml. Samples of cell suspensions were sedimented onto microscope glass slides through centrifugation, then stained with Wright's stain for assessment of cell composition. Differential counting of the detached cells revealed that more than 90% of them constituted large and 10% small monocyte cells. Volumes of 0.1 ml of the suspensions in RPMI 1640 + 5% FBS containing $3–3.5 \times 10^5$ cells were introduced into the wells and supplemented with 0.1 ml of medium or medium containing the various stimulants.

*Schistosomula*: Schistosomula of *S. mansoni* of Egyptian origin which was routinely passaged through Puerto Rican *Biomphalaria glabrata* snails, were produced by mechanical transformation of cercariae, according to Lazdins *et al.*[10] Briefly, shed cercariae were collected and concentrated, and their tails sheared off by vortexing. The cercarial bodies were now separated from the tails by centrifugation on 70% percoll (Pharmacia, Uppsala, Sweden) and incubated in Earle's BSS for 3 h at 37°C. Forty to 50 such schistosomula were then introduced into each of the test wells.

*Cytotoxicity assay*: The cells were usually incubated for 7 days, rarely up to 14 days, and once for 21 days, with or without GM–CSF, IL-3 or IFN-γ. After the preincubation period, 100 μl/well were supplanted by 100 μl of 5% FBS-supplemented RPMI 1640 medium containing the initial concentrations of GM–CSF, IL-3 or IFN-γ and 40 to 50 3 h-old, mechanically transformed schistosomula,[10] and further incubated for 48 h. Where appropriate, IFN-γ or LPS were added to the cultures. In the flat-bottomed wells, therefore, the cell to schistosomula ratio was slightly below $10^4$ : 1, whereas for the round-bottomed well plates, the ratio of monocytes to schistosomula was approximately $6–7 \times 10^3$:1.

Cytotoxicity of the macrophages was assessed by determining the percent dead schistosomula and subtracting from it the percent dead schistosomula in wells containing growth medium only (control wells). Counts of schistosomula were performed in duplicate wells, and their death was assessed by immobility, overall granular appearance and inactivity of their flame cells.

In addition to calculated averages, the readings were also graded as follows: negative (less than 5% schistosomulicidal activity), weakly positive (5–20% cidal activity), moderately positive (20–35% cidal activity) or strongly positive (with over 35% killing of the schistosomula). Stimulators/modulators at the highest concentration employed in the experiments were added in parallel to wells containing schistosomula but no cells. These wells served as controls for the possible direct deleterious effect of the materials on the schistosomula.

*Determination of tumour necrosis factor-α concentration*: TNF-α concentration was determined in the supernatants of wells of the cytotoxicity assays in order to detect a possible correlation between this concentration and the rate of anti-schistosomular toxicity. It was measured in a bioassay based on destruction of cells belonging to the L-929 line of mouse fibroma origin in proportion to its concentration. The extent of the cell destruction was read in a microplate spectrophotometer (EL309, Bio-Tek instruments, Winowski, VT, USA) as O.D. units, after staining the cells by Hemacolor and extracting the dye.[11] TNF-α concentrations (ng/ml) in the wells were ultimately determined by comparison with readings of known concentrations of human recombinant TNF-α (kindly supplied by Behringwerke).

### Results

*Well configuration*: The importance of well configuration became evident when the number of positive tests out of the total performed was counted. Thus, in flat-bottomed plates, where 4–7 repetitions were performed for each of 0, 4, 7 and 14-day cell preincubations prior to addition of the schistosomula to the wells, only in one experiment, involving 7-day preincubation groups, was there toxicity to the cultured young worms: high cidal activity (57%) in one of six preincubations with IL-3 and moderate cidal activity (20%) in one of six preincubations with GM–CSF. For the round-bottomed well plates, average results are shown in Table 1. It may be seen from the table, based both on averages and on cytotoxicity ranges, that IL-3 exerted a somewhat stronger cidal effect than did GM–CSF. This effect, though visually detectable, was statistically non-significant, as evidenced by Student's *t*-test.

*LPS and IFN-γ*: The means and standard error of means for the test groups with and without additional LPS are shown in Table 1. As seen from the table, addition of LPS produced some decrease of the

**Table 1.** Influence of various stimulators on the schistosomulicidal activity of human monocytes/macrophages

| LPS added following preincubation | Schistosomulicidal activity with co-incubating stimulant (%) | |
|---|---|---|
| | GM–CSF | IL-3 |
| – | 15.0 ± 7.0 | 19.0 ± 5.3 |
| + | 10.4 ± 3.4 | 15.9 ± 5.2 |

Subdivision of schistosomulicidal activity

| Cytotoxicity (%) | LPS added following preincubation | GM–CSF | IL-3 |
|---|---|---|---|
| Negative | – | 12/17 | 6/17 |
| | + | 10/17 | 7/17 |
| 5–20 | – | 2/17 | 4/17 |
| | + | 5/17 | 5/17 |
| 20–35 | – | 3/17 | 5/17 |
| | + | 1/17 | 4/17 |
| > 35 | – | 0 | 2/17 |
| | + | 1/17 | 1/17 |

Human monocytes at 3 × $10^5$/well, in 200 μl RPMI 1640 + 5% FBS, were preincubated for the specified times at 37°C and in presence of 7.5% $CO_2$, then LPS at 10 μg/ml was either added or not to the wells, together with 50 schistosomula of *S. mansoni* and incubated under identical conditions for an additional 48 h. Death of schistosomula was judged by non-motility of the organisms and their flame cells. Seventeen repetitions were made of the preincubations. Values are given as means ± S.E.M.

toxic effect of the cells preincubated with either GM–CSF or IL-3, which was statistically non-significant. Neither did addition of LPS to cell cultures not preincubated with GM–CSF or IL-3 essentially alter the schistosomulicidal activity of the cells.

IFN-γ, which was co-incubated with the cells at 1000 U/ml and/or was added to the cell + schistosomula cultures for the last 48 h at the same concentration, produced a very weak cidal effect (7.8% ± 4.9) in only two out of twelve experiments (involving preincubation and additional 48 h incubation). Neither did it enhance the toxicity of the cells preincubated with IL-3 or with GM–CSF.

Table 2 again shows that LPS did not enhance schistosomulicidal activity of the cells, and seemingly even suppressed it (as already indicated from Table 1). TNF-α was produced at highly enhanced levels upon incubation of the cells with LPS, but apparently these high levels had, in several cases, only a slight effect on the killing of the schistosomula (upon addition of both IFN-γ and LPS), and in other cases seemed to suppress cytotoxicity and schistosomular death. It should be pointed out that these variable findings pertained only to cells preincubated with IL-3 or GM–CSF, while IFN-γ, either preincubated with the cells, added later or combined, exerted no such cytotoxic effect.

*$N^G$MMA:* $N^G$MMA did not alter the comparatively rare cidal effects that the macrophages exerted on the 3-h schistosomula introduced into the IL-3, GM–CSF or IFN-γ containing wells, with or without the additional presence in the culture wells of LPS, IFN or both, for the last 48 h of incubation.

### Discussion

Schistosomulicidal assays have heretofore been performed either in flat-bottomed 24-well plates for adherent monocytes/macrophages,[4,12] in flat-bottomed 96-well plates, or in adherence-preventing polypropylene tubes.[6–12] Maturation of the monocytes to macrophages was performed in flat-bottomed, adherence-promoting polystyrene dishes[7] or in adherence-preventing polypropylene tubes or bags.[5,6] In the present study, the toxicity assays as well as the cell preincubation with the various materials were performed in either flat-bottomed 96-well plates for adherent cells, or in round-bottomed 96-well plates, which because of sloping of the well bottom, yielded, after several days of culturing, both adherent and non-adherent monocytes/macrophages. Taking the 7 d preincubations as the basis for comparison, only in one of twelve experiments performed in flat-bottomed well plates was there a schistosomulicidal effect, whereas in the round-bottomed well plates, 7/17 and 5/17 experi-

**Table 2.** Correlation between TNF-α production and schistosomulicidal activity of human monocytes/macrophages

| Stimulator added* | Cidal activity and TNF-α production** for preincubation of cells for 7 d with stimulatory compounds | | | | | | |
|---|---|---|---|---|---|---|---|
| | Experiment No. 1 | | | Experiment No. 2 | | | |
| | Medium | GM–CSF | IL-3 | Medium | GM–CSF | IL-3 | IFN-γ |
| None | **0** (1.1) | **0** (1.5) | **10.1** (1.2) | **0** (0.2) | **83.3** (0) | **85.1** (0.1) | **0** (N.D.) |
| LPS | **0** (2.7) | **2.9** (17.7) | **11.1** (9.3) | **0** (1.2) | **50.0** (36.3) | **5.8** (65.0) | **0** (N.D.) |
| IFN-γ | **0** (N.D.) | **8.1** (9.4) | **2.6** (2.7) | **0** (N.D.) | **81.0** (0.2) | **69.6** (0) | **0** (0) |
| LPS ± IFN-γ | **0** (14.7) | **18.4** (41.1) | **8.6** (18.5) | **0** (1.0) | **22.8** (41.7) | **8.0** (17.2) | **0** (0) |

The human monocytes in these experiments originated from two different donors. They were incubated in round-bottomed well of microtitre plates at 3 × $10^5$/well, in 200 μl RPMI 1640 + 5% FBS, at 37°C and in 7.5% $CO_2$ in air. Following incubation with the indicated materials for either 4 d or 7 d, LPS, IFN-γ or both were introduced into the wells, together with 50 schistosomula and incubated for another 48 h. Cell to parasite ratio was 6 × $10^3$ : 1. Death of schistosomula was judged as in footnote for Table 1. Levels of TNF-α were assessed in a bioassay.
* Added to culture after preincubation and co-incubated with the cells for the last 48 h of the experiment.
** Values in parentheses are TNF-α concentrations (ng/ml). Other values are the percent death of schistosomula.

ments yielded toxic effects upon GM–CSF preincubation with or without respective additional LPS, and correspondingly 10/17 and 11/17 for the equivalent IL-3 preincubations. These results strongly suggest that the non-adherent portion of the macrophages (non-adherent, purely due to the well configuration, which enabled the adherence of only a small proportion of these cells) sedimenting and concentrating in large mass in a small area of the well, is more contributory to the cidal effect than is the adherent portion, which may partly explain the negative results obtained by Remold *et al.*[5] and the moderate toxicity obtained by Ellner and Mahmoud.[4] We must take into account, however, that these groups made their readings after 24 h of co-incubation with the schistosomula and this, too, may have contributed to the low or negative cytotoxicity, because apparently 40 h of co-incubation are necessary for maximal expression of schistosomulicidal activity,[7] albeit Cottrell *et al.*[6] found that at cell to parasite ratios exceeding $5 \times 10^3 : 1$, a very high death rate of the schistosomula (81%) occurred after 24 h co-incubation. In accord with the two last mentioned works, no schistosomula-directed cytotoxicity of freshly isolated human monocytes was detected in our experiments. However, unlike the findings of James *et al.*[7] and our own, Cottrell *et al.*[6] found that non-stimulated maturation of the monocytes by itself produced high cytotoxicity after 5 days of cultivation, a process accelerated by inclusion in the growth medium of high concentrations ($10^4$ U/ml) of human recombinant IFN-γ. As already mentioned, in the present study human recombinant IFN-γ, regardless of whether preincubated with the cells *ab initio*, added to the wells for the last 48 h, or used in a combination of both protocols, essentially did not alter the negative cytotoxicity of the cells cultured without the additivies. The same was true also for preincubations containing IFN-γ with either GM–CSF or IL-3, which is contrary to the results obtained by James *et al.*[7] in similar combinations involving human recombinant IFN-γ and CSF-1. From the results shown in Table 2 and from a previous study,[13] it is clear that the cells' potential to react to stimulation (here via LPS), as expressed by TNF-α production, is dependent on co-incubation with the cytokines GM–CSF or IL-3, but not with IFN-γ.

One of the unknowns in human monocyte/macrophage toxicity towards schistosomula is the mechanism involved. In contrast to mouse peritoneal macrophage schistosomulicidal activity which is arginine dependent[14] and largely nitrite mediated,[15] supernatants from human monocytes or macrophages apparently are not toxic to schistosomula.[6] According to James *et al.*,[7] neither nitrite nor $H_2O_2$ production can account for the antischistosomula toxicity expressed by human macrophages. In the same work, TNF-α production was assessed upon IFN-γ or LPS stimulation of the cells, and it was found that LPS elevated by more than a hundred-fold TNF-α production by fresh monocytes as well as by macrophages which had been cultured in the presence of IFN-γ for 2 weeks. However, in the present study, fresh monocytes as well as 7 d macrophages did not become schistosomulicidal upon LPS stimulation despite high levels of TNF-α produced by them.

Regrettably, James *et al.*[7] did not show the possible correlation between the TNF-α levels produced by their stimulated cells and either the cidal effect of the producer cells or that of the respective TNF-contained supernatants, so their results and ours are not fully comparable. Even so, their cells produced, under the most optimal conditions, only about 3.4 ng/ml TNF-α. Taking an approximate equivalent of 20 units per nanogram (calculated from data shown in the most recent 'Genzyme' company catalogue), this would mean slightly more than 68 units of TNF-α. According to James *et al.*,[8] this amount of exogenous TNF-α added to the culture medium yielded only background, or slightly higher levels of schistosomulicidal activity. Hence, it is doubtful, although not entirely impossible, that a micro-concentration of this material could build up and exert a toxic effect on the schistosomula at the interface of contact between the macrophages and the parasites, as has been speculated by us regarding the potential schistosomulicidal effect of macrophage-derived lysozyme.[16]

Finally, if the positive results obtained by Cottrell *et al.*,[6] with or without stimulation of the cells, and by James *et al.*[7] upon their stimulation, pertain to all their blood donors and not just the positively reacting ones, then the present findings are contrary to theirs in indicating that the schistosomulicidal effect of the cells from different donors may fluctuate widely, from completely negative to over 80% killing in round-bottomed well plates, *vs.* almost complete ineffectiveness in the flat-bottomed well plates. If the divergent results obtained by us are not merely an artefact of differing techniques, they could reflect hitherto unknown inherent differences in the blood used. They could, for instance, indicate factors linked to primary or secondary blood group markers, or to major MHC-class I or II antigens. They might, on the other hand, reflect the occurrence, in the small proportion of reactive bloods, of nonspecific prestimulation of the monocytes by unknown factors.

Relying on lack of toxic nitrites production by human monocyte-derived macrophages[7] and on our own data which showed that an arginine metabolism inhibitor did not alter the macrophage toxicity and that high levels of TNF-α did not enhance the anti-schistosomula toxic effect, it may be theorized that human monocyte-derived macrophages do not participate in protecting against schistosome invasion, but merely in killing the parasite's egg-embryo within the schistosome-induced granuloma.

The present study was intended to resolve some of the contrasting results and conclusions regarding human macrophage toxicity toward larval stages of schistosomes. Our findings, however, indicate very little such toxic reactivity, and thus far the mechanism whereby schistosomula are killed is uncertain, even in studies where an efficient schistosomulicidal effect has been shown.

This is not so in the case of mouse macrophages, where a dependency on arginine metabolism was first confirmed in a former work,[14] to be followed by the implication of nitrites as the effector molecules.[15]

### References

1. Seger M, Gold D, Lengy J, Pauli H, Keisari Y. On the interactions between macrophages and developmental stages of *Schistosoma mansoni*: effect of muramyl tripeptide ethanolamine (MTP–PE) treatment on mice survival and the generation of schistosomulicidal macrophages. *Parasite Immunol* 1992; **14:** 355–369.
2. James SL, Sher A. Cell-mediated immune response to schistosomiasis. *Curr Topics Microbiol Immunol* 1990; **155:** 21–31.
3. Capron M, Capron A. Rats, mice and men—models for immune effector mechanisms aginst schistosomiasis. *Parasitol Today* 1986; **2:** 69–75.
4. Ellner JJ, Mahmoud AAF. Killing of schistosomula of *Schistosoma mansoni* by normal human monocytes. *J Immunol* 1979; **123:** 949–951.
5. Remold HG, Mednis A, Hein BA, Caulfield JP. Human monocyte-derived macrophages are lysed by schistosomula of *Schistosoma mansoni* and fail to kill the parasite after activation with interferon gamma. *Am J Pathol* 1988; **131:** 146–154.
6. Cottrell B, Pye C, Butterworth A. Cytotoxic effects *in vitro* of human monocytes and macrophages on schistosomula of *Schistosoma mansoni*. *Parasite Immunol* 1989; **11:** 91–104.
7. James SL, Cook KW, Lazdins JK. Activation of human monocyte-derived macrophages to kill schistosomula of *Schistosoma mansoni in vitro*. *J Immunol* 1990; **145:** 2686–2690.
8. James SL, Glaven J, Goldberg S, Meltzer M, Pearce E. Tumour necrosis factor (TNF) as a mediator of macrophage helminthotoxic activity. *Parasite Immunol* 1990; **12:** 1–13.
9. Treves AJ, Yagoda D, Haimovitz A, Ramu N, Rachmilewitz D, Fuks Z. *J Immunol Meth* 1980; **39:** 71–80.
10. Lazdins JK, Stein MJ, David JR, Sher A. *Schistosoma mansoni*: rapid isolation and purification of schistosomula of different developmental stages by centrifugation on discontinuous density gradients of percoll. *Exper Parasitol* 1982; **53:** 39–44.
11. Keisari Y. A colorimetric microtiter assay for the quantification of cytokine activity on adherent cells in tissue culture. *J Immunol Meth* 1992; **146:** 155–161.
12. James SL, Sher A, Lazdins JK, Meltzer MS. Macrophages as effector cells of protective immunity in murine schistosomiasis. II. Killing of newly transformed schistosomula *in vitro* by macrophages as a consequence of *Schistosoma mansoni* infection. *J Immunol* 1982; **128:** 1525–1540.
13. Robin G, Markovich S, Athamna A, Keisari Y. Human recombinant granulocyte-macrophage colony-stimulating factor augments viability and cytotoxic activities of human monocyte-derived macrophages in long-term cultures. *Lymphok Cytok Res* 1991; **10:** 257–263.
14. Malkin R, Flescher E, Lengy J, Keisari Y. On the interactions between macrophages and developmental stages of *Schistosoma mansoni*: The cytotoxic mechanisms involved in macrophage-mediated killing of schistosomula *in vitro*. *Immunobiology* 1987: **176:** 63–72.
15. James SL, Glaven J. Macrophage cytotoxicity against schistosomula of *Schistosoma mansoni* involves arginine-dependent production of reactive nitrogen intermediates. J Immunol 1989; **143:** 4208–4212.
16. Flescher E, Keisari Y, Lengy J, Gold D. On the possible schistosomulicidal effect of macrophage-derived lysozyme. *Parasitology* 1991; **103:** 61–64.

**Received 21 March 1994; accepted in revised form 2 May 1994**
